# Multiple sclerosis diagnosis in south Asia: fundamentals first

**DOI:** 10.1016/j.lansea.2026.100780

**Published:** 2026-05-11

**Authors:** The Lancet Regional Health – Southeast Asia

Multiple sclerosis (MS), a chronic auto-immune demyelinating disorder of the central nervous system, is an important cause of young-onset neurological disability, with the average age at diagnosis being 30–33 years across all six WHO regions. An estimated 2.9 million people are living with MS worldwide, with prevalence shown to be increasing. In south Asia, demographic patterns are similar to global trends, including a predominance among women. A primary clinical challenge in south Asia can be in establishing a timely and certain diagnosis of MS across its diverse healthcare contexts.

Early diagnosis and intervention in MS are crucial but challenging, with symptoms differing vastly in onset, presentation, and resolution. For example, early in the disease course, individuals can have visual symptoms, fatigue, motor deficits, and sensory symptoms such as numbness or tingling. This, together with fragmented referral pathways, elevates the potential for misdiagnosis-which is further exacerbated by the lack of region-specific data on MS clinical presentation. Although prognosis of MS varies, being determined by numerous factors, early initiation of effective disease modifying therapies can slow progression.

To that end, the past two decades have seen an evolution in MS diagnosis, with increasing reliance on magnetic resonance imaging (MRI) to support clinical presentation. The 2001 landmark McDonald criteria established MRI-based evidence as central to diagnosis, enabling earlier identification of MS through evidence of lesions disseminated in space and time within the central nervous system, alongside clinical symptoms. The 2024 revisions further heighten the emphasis on imaging relative to previous iterations. However, these criteria were developed within clinical and radiological contexts that assume ready access to high-quality imaging, longitudinal follow-up, and a narrower spectrum of differential diagnosis than met in south Asia.

MRI interpretation can be a source of misdiagnosis in south Asia where the recommended standardised MRI protocols may be harder to implement due to limited availability and access to MR imaging and specialist neuro-radiology review-thus limiting validity of findings. Advanced sequences such as susceptibility-sensitive imaging, which are increasingly used in higher-income settings, are less available in low-income and middle income (LMIC) contexts. Furthermore, when it may be required to establish dissemination in lesions over time, serial imaging is required. In many south Asian settings, access to repeat MRI is limited by cost and availability, reducing feasibility of a longitudinal assessment. Moreover, although imaging markers such as the central vein sign and paramagnetic rim lesions have shown promise in improving diagnostic sensitivity for MS, evidence supporting their use is largely derived from cohorts in higher income settings, with limited validation in south Asian populations.

Imaging findings must also be interpreted within broader considerations. Demyelinating disorders such as myelin oligodendrocyte glycoprotein associated disease (MOGAD) and aquaporin-4 antibody positive neuromyelitis optica spectrum disorder (AQP4+ NMOSD) can have overlap in imaging with MS and are relatively more prevalent in Asian populations. Access to confirmatory investigations such as antibody testing for aquaporin-4 and MOG are limited. The picture can be further complicated by the higher prevalence of infectious conditions in the region with clinical or radiological features that closely mimic MS. These include central nervous system tuberculosis and neurocysticercosis. At first presentation, the distinction between infectious and inflammatory pathology may not be apparent. Clinical identification of MS remains challenging in these circumstances. In addition, the absence of a large body of local data and higher prevalence of conditions mimicking symptoms of MS can complicate differential diagnosis.

May 30 marks World MS Day, with the 2026 theme “My MS Diagnosis” emphasising timely, accurate diagnosis for everyone living with MS. There is a clear need for diagnostic approaches to MS that reflect local realities, resources and clinical presentations. This will require strengthening local research in this sphere. There is a dearth of registry data in South Asia, which has been stated to be a key research priority by the MS International Federation. This issue of *The Lancet Regional Health – Southeast Asia* includes findings from Bhatia and colleagues on demyelinating disorders of the central nervous system in the Indian population, and we hope for additional contributions from the region in the coming years. Attention must remain on the fundamentals—early and accurate diagnosis, appropriate imaging, and region-specific evidence.

